# Agro-productivity amidst environmental degradation and energy usage in Nigeria

**DOI:** 10.1038/s41598-021-98250-y

**Published:** 2021-09-23

**Authors:** Bosede Ngozi Adeleye, Praise Daramola, Ademola Onabote, Romanus Osabohien

**Affiliations:** 1 Department of Economics and Development Studies, Covenant University, Ota, Nigeria; 2 Regional Centre of Expertise (RCE) Ogun, Covenant University, Ota, Nigeria; 3 Centre for Economic Policy and Development Research (CEPDeR), Covenant University, Ota, Nigeria; 4 Department of Economics, Landmark University, Omu-Aran, Nigeria

**Keywords:** Climate sciences, Environmental sciences, Environmental social sciences

## Abstract

This study revisits the 2030 United Nations Sustainable Development Goal (SDG) 2 which aims to “*end hunger, achieve food security, improve nutrition and promote sustainable agriculture*” by highlighting the impact of environmental degradation (proxied by carbon emissions) and non-renewable energy on agro-productivity in Nigeria. Using annual time series data from 1980 to 2018, the study engages the Johansen cointegration and impulse response functions (IRFs) techniques within the vector autoregressive (VAR) framework. Evidence reveals that carbon emissions significantly reduce agro-productivity by 0.23% while non-renewable energy boosts agro-productivity by 5.38%, on average, *ceteris paribus*. Other results reveal that domestic credit, rural population and arable land exert asymmetric effects. These outcomes are consistent and align with a priori expectations. Policy recommendations are discussed.

## Introduction

This paper revisits the response of agricultural productivity to environmental degradation and nonrenewable energy by presenting empirical findings which fill a gap in the literature. This research takes a new perspective and highlights findings on whether carbon emissions (proxy for environmental degradation) and nonrenewable energy negatively impact food production (proxy for agro-productivity). That is, will the use of energy-intensive inputs (fertilizers, mechanization and irrigation) improve yields and consequently the ability to grow enough food? Conclusions reveal, *inter alia*, that in the long-run, environmental degradation is a significant negative predictor of agro-productivity while nonrenewable energy shows an increasing effect. Supportively, findings from the impulse response show that agriculture reacts negatively to positive shocks from emissions while it responds positively to shocks from energy. In essence, both variables are crucial determinants of agro-productivity that cannot be ignored in the quest to achieve the 2030 United Nations Sustainable Development Goal (SDG) 2. Hence, to adequately feed its growing population, the country needs to take urgent measures to improve the usage of non-renewable energy sources and control the level of carbon emissions These are significant contributions to the agriculture literature and provides the justification for engaging in this study.

In Africa, the agricultural sector experiences severe challenges arising from climate change due to rising level of “greenhouse” gases (GHGs) and carbon dioxide (CO2) emissions^15,27^. This is because the agricultural sector relies majorly on rain-feed and good vegetation. Again, the sector is highly sensitive to environmental conditions. Agriculture is known to be the base of developing economies, especially, countries in Africa where it constitutes about 50% of the total employment and livelihood^36^. Farming method in this region is mainly at a subsistence level. Thus, the effects of emissions and climatic shocks are predicted to result in low productivity, shortage of food and welfare of the farmers^37^. In addition, emission-related risks and other agricultural issues may negatively affect productivity, if not properly tamed. Recently, the continent has witnessed severe reduction in natural resources, constraining the productivity of the agricultural sector^39^. Increasing heat intensity and variations in the pattern of rainfall also have direct impact on agricultural productivity and an indirect impact due to the availability of water rewired for irrigation. Estimates from Intergovernmental Panel on Climate Change (IPCC) showed that crop yields have reduced from 10 to 25% and this reduction will worsen by 2050 from climate change effects^39^.

Environmental degradation resulting from carbon emissions and “greenhouse” gases (GHGs) increase heat intensity^32^ leading to large proportion of the reduction of agricultural productivity^18^. This is because climate change and other emission indicators release varieties of poisons that harmfully affect the ecosystem, human well-being^13,19,20,22,48^ and have resulted in the depletion of soil nutrients as an aftermath of heat intensity^18^. On the usage of nonrenewable energy which are non-replaceable and ironically are vital reactors for economic growth, several energy-agriculture studies^5,24,26,30,34,35,41^ elaborate the positive relationship between agricultural production and the energy embodied in agricultural inputs (e.g. in the manufacture and transport of fertilizers and tractors). These studies show that when high-input agriculture substitutes energy in the form of fuels, fertilizer and other inputs for land and labour, the result is an increase in agro-yield and energy intensity.

Africa is projected to be the worst impacted by climate change^21^. The reason is because, the continent is predominantly agrarian with heavy reliance of its population on farm and agriculture linked off-farm income for survival^11,38^. Though, a less contributor to global warming, Africa remains one of the most susceptible to climate change due low economic development, high dependence on natural resources and low technological advancement^33^. The impact of climate represents a further threat to the region’s multi-faceted socio-economic challenges. Furthermore^46^, conclude that carbon emissions which triggers climate change affects agro-productivity directly through its effect on crop yields, crop pests and diseases, and soil fertility and water-holding properties. In the same vein^45^, suggest that climate change can also indirectly affect agro-productivity through its effect on economic development, income distribution and agricultural demand. That is, erratic climate conditions negatively affect the solidity of crop yields.

Similarly, emissions harmfully affect access to food as agricultural production declines leading to rising food price and decline in purchasing power. In spite of agriculture being the predominant activity, Africa currently has the highest number of chronic undernourished population with exposure to the highest degree of instability in food production due to extreme dependence on pollution emitting energy services and climate variability^7^. On the devastating impact of environmental degradation (proxied by climate change) on agro-productivity, accumulation of radioactive GHGs in the air usually cause enhanced greenhouse effect^1^. The subsequent damage caused by the surrounding vegetation's consumption of toxic carbon emissions can affect plant quality and aesthetic value which reduce their economic value^49^. Also, when carbon dioxide sinks in the atmosphere the resulting water may become harmful to vegetation^9,23^. Thus, aligning with the 2030 United Nations Sustainable Development Goal (SDG) 2 which aims to *“end hunger, achieve food security, improve nutrition, and promote sustainable agriculture”,* this study revisits the issue of agro-food productivity amidst threatening environmental degradation and the usage of non-renewable energy sources.

As the most populous black country in the world (https://worldpopulationreview.com/country-rankings/richest-african-countries) with 193million people, Nigeria occupies the seventh position as the world’s 17th biggest emitter of greenhouse gases in 2015 and the second highest in Africa after South Africa (https://www.carbonbrief.org) Hence the focus on Nigeria is germane. According to the International Monetary Fund (https://www.imf.org/external/datamapper/profile/NGA), Nigeria is the largest economy in Africa with an average GDP of USD514 billion followed by Egypt with USD394.28 billion and South Africa at USD329.53 billion. Though heavily dependent on crude oil exports, efforts are intensified to diversity oil-dependence to agricultural sector. Statistics from the National Bureau of Statistics (https://www.nigerianstat.gov.ng/)reveal that the country’s agro-sector is the largest single economic sector by contribution to real GDP growth as the sector grew by 10.6% year-on-year in nominal terms during the second quarter of 2018.

To probe the discourse, annual time series data from 1980 to 2018 comprising six variables is engaged. The dependent variable is food production. The main explanatory variables are carbon emissions per capita (proxy for environmental degradation) and nonrenewable energy per capita. The control variables are domestic credit, arable land, and rural population growth. All variables are sourced from ^50^. The study engages the Johansen cointegration techniques to explore if any long-run relationship exists and the impulse response function (IRF) which describes the reaction of one variable to the innovations in another variable in the system, while holding all other shocks equal to zero^4,29,40,47^. This empirical approach makes the study holistic to ensuring a critical examination of its core arguments. The rest of the paper is structured as follows: section “Brief literature review” discusses the literature; section “Data and empirical model” outlines the data and empirical model; section “Results and interpretations” discusses the results, and section “Discussion” concludes.

## Brief literature review

Various studies^8,31,43,44^ using different approaches, provide evidence on the impact of emission indicators, renewable energy, climate change and global warming impact on agricultural productivity across the globe and its mitigating mechanisms such as social protection among others^28,37^. The fact is that, climate change impacts on all parts of agribusiness, and there are huge indications that increased global warming will result to a shift in seasonality and the intensity in precipitation that will intensify the weakness of the agricultural sector^18^. Some of the major components of climate change is carbon and greenhouse gas emissions, which negatively affect agricultural productivity. In line with this, the study by ^37^ using the fixed and random effects approaches, examined how gas emissions affect crop production in the Economic Community of West African States (ECOWAS) sub-region. The study found that, carbon emissions, especially, greenhouse gases and other climate change components reduce agricultural productivity by about 0.13%. Therefore, giving this, to prevent the impact of climate change on productivity and health, there is the need for mitigating policies and programmes such as the social protection^27,37^.

Mitigating environmental degradation becomes necessary because by 2050, the global population may rise to about 9 billion which may likely increase global warming and worsen the agricultural systems^16^. In another study^17^, using and in-depth review of the literature and stylised facts, asserted that farming, or in general, the agricultural system is highly controlled by climatic conditions. In this wise, unfavourable weather and climate induced by human efforts such as carbon emissions will reduce agricultural yields, which pose a threat to food security and human health. On the other hand, while farmers are most adaptable in handling the menace of weather and climatic conditions yearly, there is however a necessary condition to the adaption of country-specific climate reforms in the form of proven indigenous farm practice and personal experience^17^.

In terms of energy consumption, agricultural development through farm mechanisation has contributed a lot^44^. Taking a clue from Asian studies^44^, examined the linkage between some emission indicators (C02 and energy usage) and agricultural productivity (measured by cropped yield) and other covariates such as enhanced seed distribution, overall food grains and adequacy of water (for the period 1987–2017). The study applied the ARDL econometric approach and found that, in the long-run, cropped area, consumption of energy, per capita GDP and the adequacy of significant and positively related to emission indicators. Across the global, especially, in the last two decades, outside the energy problems, carbon and the greenhouse gas emissions, are some of the most prominence causes of global warming, which have gained prominence consideration in the literature^37^. The study of^42^ examined how energy usage of energy and emissions of greenhouse gases affect agricultural productivity, using the cucumber farm in some greenhouses in Yazd district of Iran. The study found that energy use efficiency and productivity were 0.10 and 0.12 kg, respectively. In addition, diesel fuel and electricity power had the biggest contributions in the total energy input. Estimates form the economic analysis showed that diesel fuel had a significant impact on agriculturally productivity in Iran. In this wise, the study estimated the amount of greenhouse gas emissions at about 82,724 kg CO2eq ha1, where diesel fuel had the highest emission (61%). These studies show that to achieve sustainable development in agriculture, it is highly imperative to manage energy usage and gas emissions in all production processes to mitigate the impact of climate variation.

## Data and empirical model

### Variables

Annual time-series data obtained from ^50^ for a period ranging from 1980 to 2018, thus spanning 39 years is used in the study. The data used include food productivity (*FDPRD*) which is the dependent variable while carbon emissions per capita (*CO2PC*) and nonrenewable energy per capita (*ENUPC*) are the main explanatory variables. Arable land (*ARL*), rural population growth (*RUGP*), domestic credit (*CR*) and are the control variables.

On a priori expectations, *CO2PC* captures environmental degradation which hampers farm yield and output. It is predictable that as the environment worsens it impacts negatively on agro-productivity. Hence, a negative coefficient is expected. *ENUPC* captures more energy usage either for agricultural use which are direct inputs to aid productivity. A positive coefficient is expected. Similarly, *ARL* and *RUGR* are natural enablers of agro-productivity. The impact of *CR* may be asymmetric. (High) low cost of credit/loans creates the (dis)incentive to borrow by farmers which may (inhibit) encourage the purchase of farm implements, improved seedlings, and the acquisition of more expanse of land leading to (decrease) increase in agro-productivity. Given the expressed scenarios, a (negative) positive coefficient is expected. Table 1 presents a tabular representation of the variables, their measurements and expectations.

**Table 1:** Variables, measurement and expectations.

Variables	Description of Variable	Measurement	Expectations
$FDPRD$	Food Productivity	Kilogram per hectare	Not Applicable
$CO2PC$	Carbon emissions per capita	metric tons per capita	Negative
$ENUPC$	Nonrenewable energy use per capita	kg of oil per capita	Positive
$CR$	Domestic credit	% of GDP	Positive/Negative
$RUGR$	Rural population growth	% of total population	Positive
$ARL$	Arable land	% of land area	Positive

Source: Authors’ Compilations.

### Model specification

This study extends ^5^ and ^37^ to rationalise if, given other factors that affect agro-productivity, carbon dioxide emissions (proxy for environmental degradation) and nonrenewable energy usage significantly impact food production in Nigeria. The implicit model expressing the relationship is stated as:

1 $$FDPRD = f\left( {CO2PC, ENUPC, CR, RUGR,ARL} \right)$$

where the variables are as defined in Table 1. Equation (1) expresses agro-productivity as an implicit function of the endogenous variables, the explicit form of which is specified in its logarithmic form as:

2 $${\text{ln}}FDPRD_{t} = b_{0} + b_{1} {\text{ln}}CO2PC_{t} + b_{2} {\text{ln}}ENUPC_{t} + b_{3} {\text{ln}}CR_{t} + b_{4} {\text{ln}}RUGR_{t} + b_{5} {\text{ln}}ARL_{t} + e_{t}$$

where $b_{0}$ is the intercept, $b_{1}$ to $b_{5}$ are the parameters to be estimated and $v_{t}$ is the error term.

### Estimation techniques

The study employs two techniques to analyse the study objectives: (1) the Johansen cointegration technique within the vector autoregressive (VAR) model and (2) the impulse response function (IRF). The Johansen cointegration test which explores co-integration is used to express the dynamic relationship amongst the variables of interest, and to observe the short- and long-run dynamics of the model. Hence, co-integration equation with VAR (*k*) is given as:

3 $$Y_{t} = { }\rho + \varphi_{1} Y_{t - 1} + \varphi_{2} Y_{t - 2} + \cdots + { }\varphi_{k} Y_{t - k} + \varepsilon_{t}$$

where $Y_{t}$ = vector of endogenous variables; $\rho$ = is the constant term; $\varphi_{1} - \varphi_{k}$ = matrices of the coefficient parameter; $Y_{t - 1}$ = vector of the lagged endogenous; $\varepsilon_{t}$ = vector of the error term.

Equation (3) is expressed in matrix form as:

4 $$\left[ {\begin{array}{*{20}c} {Y_{t} } \\ {Y_{t - 1} } \\ \cdot \\ \cdot \\ \cdot \\ {Y_{t - k} } \\ \end{array} } \right] = \rho + \left[ {\begin{array}{*{20}c} {\varphi_{1} } & {\varphi_{2} } & \cdot & \cdot & \cdot & {\varphi_{k} } \\ 1 & 0 & \cdot & \cdot & \cdot & 0 \\ \cdot & \cdot & \cdot & \cdot & \cdot & \cdot \\ \cdot & \cdot & \cdot & \cdot & \cdot & \cdot \\ \cdot & \cdot & \cdot & \cdot & \cdot & \cdot \\ 0 & 0 & \cdot & \cdot & \cdot & 0 \\ \end{array} } \right] \cdot \left[ {\begin{array}{*{20}c} {Y_{t - 1} } \\ {Y_{t - 2} } \\ \cdot \\ \cdot \\ \cdot \\ {Y_{t - k} } \\ \end{array} } \right] + \varepsilon_{t}$$

where $y_{t}$ represents the vector of the endogenous variables $\left( {FDPRD,CO2PC, ENUPC, CR, RUGR,ARL} \right)^{^{\prime}}$ which are all stationary at *I*(1) and $\varepsilon_{t}$ represents the vector of shocks. Next, it is necessary to determine the status of the long run relationship between the variables.

The Johansen cointegration technique is used to test this. The Johansen cointegration is a VAR process and but works only when the all variable is integrated of the same order; for example, I(1). It uses the Trace statistics and Max-Eigen statistic to determine the existence of a long run relationship among the variables. Another analysis conducted is the impulse response function, which aims to illustrate the reaction of food production to a shock from one standard deviation in the impulse of other variables in the model^4^. The IRF on the other hand simulates the effect of a shock to one variable in the system on the conditional forecast of another variable. According to^14^ there are numerous interesting applications in which a researcher might be interested in calculating an impulse response function. Without any loss of consistency, this approach accommodating interactions among the variables in the system. This view is also supported by^25^ who affirms that a further advantage of the IRF is that it can also be used to evaluate the effectiveness of a policy change on the target variable(s). The generalized impulse response function of $y_{t}$ at horizon $h$ is defined as:

5 $$IRF\left( {h, \delta , I_{t - 1} } \right) = E\left[ {y_{t + h} |e_{t} = \delta ,I_{t - 1} } \right] - E\left[ {y_{t + h} |I_{t - 1} } \right]$$

where $\delta$ is the one time exogenous shock. Equation (5) explains that the impulse response function equals the expected value of current and future values of an endogenous variable given the shock and past information minus the expected value of the endogenous variable given past information. In order words, it is the effect of the shock on the current and future values of the endogenous variable.

## Results and interpretations

### Summary statistics and correlation analysis

The analytical procedure begins with the examination of the properties of the variables such as measures of central tendency and relative associations through the summary statistics and correlation analysis. Summary statistics are employed to understand the properties of each variable from their maximum and minimum values, mean and standard deviation while the correlation analysis is performed to appraise the association among the variables. In specific terms, correlation explains the strength of the linear relationship between variables. The outcomes of both procedures are shown in Table 2.

**Table 2:** Summary statistics and correlation analysis.

Variable	*FDPRD*	*CO2PC*	*ENUPC*	*CR*	*RUGR*	*ARL*	
Mean	77.110	0.611	715.534	9.237	1.415	33.793	
Standard Dev	31.736	0.184	36.176	3.575	0.323	6.218	
Minimum	29.970	0.326	665.436	4.958	0.939	18.079	
Maximum	125.770	0.928	798.630	19.626	2.272	40.625	
*Pairwise Correlation Analysis*							
*FDPRD*	1.000						
*CO2PC*	− 0.326*	1.000					
*ENUPC*	0.824***	0.052	1.000				
*CR*	0.673***	0.105	0.603***	1.000			
*RUGR*	− 0.763***	− 0.212	− 0.827***	− 0.635***	1.000		
*ARL*	0.875***	− 0.444***	0.597***	0.445***	− 0.539***	1.000	

Source: Authors' Computations.

*FDPRD* food production, *CO2PC* carbon dioxide emissions, *ENUPC* nonrenewable energy per capita, *CR* domestic credit by banks, *RUGR* rural population growth, *ARL* arable land.

*, and ***Denote statistical significance at the 1% and 10% levels, respectively.

Table 2 shows that the average level of agricultural production in Nigeria is approximately 77.1 percent. With arable land space of about 34 percent, availability of private sector credit at 9 percent of GDP, and rural population 1.4 percent of total population, energy use per capita is approximately 715 units. Also given emissions indicators; the average of carbon emissions over the time period was approximately 0.6 metric ton per capita. From the lower panel, the correlation matrix shows that both carbon emissions and rural population exhibit a statistically significant negative association with agro-food production while the rest variables show statistically significant positive relations.

### Unit root test results

Before variables are used in regression analysis, it should be clarified that they have averages and variance whose distribution is time-independent. This study used the Augmented Dickey Fuller (ADF) and Philip-Perron (PP) tests to check this. Table 3 shows that all variables were stationary at first difference for ADF and PP testing at 5 percent significance level, although, the average level of agricultural production shows significance at 10 percent for the ADF test. Following this evidence, the study showed that the classic approach to the ordinary least squares (OLS) procedure could yield false results if adopted. Hence, the model is estimated using the vector autoregressive (VAR) approach.

**Table 3:** Unit root test results.

Variables	Augmented Dickey-Fuller		Phillips-Perron		
	Test Statistic	5% CV	Test Statistic	5% CV	Outcomes
*FDPRD*^*a*^	− 3.270*	− 3.5403	− 7.952***	2.9434	I(1)
*CO2PC*	− 5.640***	− 2.9540	− 5.640***	2.9540	I(1)
*ENUPC*	− 4.451***	− 2.9604	− 7.547***	2.9540	I(1)
*CR*	− 4.879***	− 2.9484	− 14.249***	2.9434	I(1)
*RUGR*	− 6.421***	− 2.9434	− 6.532***	2.9434	I(1)
*ARL*	− 3.926***	− 2.9484	− 6.000***	2.9434	I(1)

Source: Authors' Computations.

*FDPRD* food production, *CO2PC* carbon dioxide emissions, *ENUPC* nonrenewable energy per capita, *CR* domestic credit by banks, *RUGR* rural population growth, *ARL* arable land.

*, ** and ***Denote statistical significance at the 1%, 5%, and 10% levels, respectively; ^a^Stationary with trend.

### Johansen cointegration and normalization results

Consequent upon the first-order integration *I*(1) of the series at the 1% and 10% significance levels, the study proceeds to perform the Johansen cointegration test and the results are shown in Table 4. From the upper panel, the optimal model as determined by the Trace and Max-Eigen statistics reject the null hypothesis of no cointegrating equation from two standpoints. The first is rejected at the 1% level given that the respective test statistics, 120.992 and 55.065 are higher than their respective critical values, 95.753 and 40.077 and the second is rejected at the 5% level with 4.114 higher than 3.841. In other words, the result indicates that the Mackinnon-Haug-Michelis *p*-value cannot reject the null hypothesis of no cointegrating relationship at the 1% and 5% levels, respectively. Hence, the foregoing shows the feasibility of long-run relationship among the variables in the model.

**Table 4:** Johansen cointegration rank test and normalization results.

Cointegrating Equations	*Trace Test*			*Max-Eigen*		
	Statistic	5% CV	Prob.**	Statistic	5% CV	Prob.**
None *	120.9928	95.75366	0.0003	55.06585	40.07757	0.0005
At most 1	65.9269	69.81889	0.0983	22.87238	33.87687	0.5402
At most 2	43.05452	47.85613	0.1313	20.61817	27.58434	0.2999
At most 3	22.43635	29.79707	0.2749	10.37763	21.13162	0.7086
At most 4	12.05872	15.49471	0.1541	7.94424	14.2646	0.3844
At most 5 *	4.114484	3.841466	0.0425	4.114484	3.841466	0.0425
*Normalized Cointegrating Coefficients*						
InFDPRD + 0.234lnCO2PC − 5.376lnENUPC + 0.936lnCR + 0.785lnRUGR − 1.080lnARL t-stat (1.968) (− 5.574) (7.978) (2.409) (− 5.754)						

Source: Authors' Computations.

*Denotes rejection of the hypothesis at the 0.05 level; **MacKinnon-Haug-Michelis (1999) probability values.

*CV* critical value, *FDPRD* food production, *CO2PC* carbon dioxide emissions, *ENUPC* nonrenewable energy per capita, *CR* domestic credit by banks, *RUGR* rural population growth, *ARL* arable land.

The lower panel of Table 4 shows the normalized cointegration (long-run) coefficients expressed in implicit form. The results show that all the explanatory variables are significant at the 1%, 5% and 10% levels. Explicit evidences from the result suggest that environmental degradation (*CO2PC*) poses a threat to agro-productivity in Nigeria. That is, in the long-run a percentage change in carbon emissions induces a 0.23 percentage decrease in agro-productivity at the 10% level, on average, *ceteris paribus*. This outcome aligns with expectations and not unconnected to the fact that Nigeria is the world’s 17th emitter of carbon dioxide and second largest in Africa after South Africa. The negative impact on agro-productivity only affirms the danger and effects on climate on agriculture and food security in general as it relates to Nigeria. The failure to put measures in place that will curb the level of emissions will result in poor vegetation with adverse consequences on food supply in the long-run. The devastation impact of carbon emissions to human life and vegetation is replete in the empirical literature^2,8,17,18,44,51^. Contrarily, nonrenewable energy (*ENUPC*) shows a statistically significant direct relation to agro-productivity. In explicit terms, a percentage change in non-renewable energy usage induces 5.38 percentage increase in agro-productivity at the 1% in the long-run, on average, *ceteris paribus*. This outcome fits expectations and the reality of Nigeria’s agrarian sector which thrives on the use of fertilizers, tractors and heavy machineries that are powered by non-renewable energy sources like crude petroleum, coal and gases which the farmers use regularly for tilling the ground, planting, harvesting and processing of crops^16^. Several studies show the direct relationship between agricultural production and energy-induced agro-implements and inputs^24,26,30,41^. Other readily available evidences reveal that domestic credit (*CR*) exerts an inverse relation to agro-productivity at the 1% significance level. Findings suggest that in the long-run, a percentage change in domestic credit leads to about 0.94 percentage drop in agro-productivity, on average, *ceteris paribus*. This outcome is not unexpected given the high lending rate charged by financial intermediaries. Despite efforts to channel funding to agriculture by various governments in Nigeria through several schemes such as Nigerian Agricultural and Co-operative Bank (NACB) established in 1972, the Agricultural Credit Guarantee Scheme Fund (ACGSF) in 1977, the Commercial Agriculture Credit Scheme (CACS), Nigerian Agricultural Insurance Corporation (NAIC) in 1987, the Nigerian Agricultural Co-operative and Rural Development Bank (NACRDB) in 2000 and the Agricultural Credit Support Scheme (ACSS) in 2006, the financial sector sees the agro-sector as highly volatile and shy away from lending to farmers or discourage farmers through high lending interest rates^5,10^. For instance, in an attempt to boost the economy by making credit cheaper, the Central Bank of Nigeria (CBN) Monetary Policy Committee (MPC) at the 21–22 September 2020 meeting decided to cut its monetary policy rate (MPR) by 100 basis points from 12.50 to 11.50% marking the second cut so far in year 2020 and the lowest since February 2016. But this move is yet to translate into cheaper credit to farmers required to boost agro-productivity. In other words, our results provide evidence of the inadequacy of finance required to spur considerable agricultural output required for food sustainability in Nigeria^10^. The outcome on rural population (*RUGR)* contradicts expectations. The coefficient which is statistically significant at the 5% level shows that in the long-run a percentage change in rural population leads to a 0.79 percentage decline in food-productivity, on average, *ceteris paribus*. The most plausible explanation for the inverse relation can be linked to the poor state of rural development in Nigeria. The lack of basic infrastructures may be driving movement to the cities which implies migration of labour from agriculture creating serious distortions in the agrarian sector and overall food supply of the country^6,12^. Lastly, the coefficient of arable land (*ARL*) conforms with expectations. It shows that at the 1% significance level, a percentage change in arable land yields a 1.08 percentage increase in agro-productivity, on average, *ceteris paribus*. Similar to Osabohien et al*.* (2020) more arable land allocated to individuals, small- and large-scale farmer will boost food production.

### Diagnostics results

Before proceeding with the impulse response functions (IRFs) analyses, it is imperative to perform diagnostics on the underlying VAR model to ascertain that the model passes all mandatory diagnostics. The results shown in Table 5 confirm that at the 5% significance level, the model does not suffer from serial correlation and heteroscedasticity in addition to having normally distributed errors.

**Table 5:** VAR diagnostics results.

Test	Stat (*p*-value)	Decision
LM Serial Correlation	46.911 (0.105)	No higher-order serial correlation
White Heteroscedasticity	553.081 (0.064)	No heteroscedasticity
Jarque Bera Normality	0.725 (0.696)	Evidence of normality

Source: Authors' Computations.

Diagnostics computed at lag 2.

### Impulse response function results

Since the impulse response function (IRF) explains the reaction of an endogenous variable to one of the innovations in the vector autoregression (VAR) system, it becomes imperative to describe the progression of the variable of interest (agro-productivity) over a specified time horizon after a shock to other variables in the VAR system^28^. IRF is an essential tool in empirical and policy effectiveness analysis, hence, our reasons for its incorporation. So, consequent upon good diagnostics, we proceed to analyse the response of agro-productivity to one standard deviation shock from each explanatory variable and the results are shown in Table 6.

**Table 6:** Response of lnFDPRD to one standard deviation shock.

Period	lnCO2PC	lnENUPC	lnCR	lnRUGR	lnARL
1	0.0000	0.0000	0.0000	0.0000	0.0000
2	− 0.0015	0.0068	− 0.0130	− 0.0096	− 0.0077
3	− 0.0156	0.0075	− 0.0112	− 0.0203	− 0.0003
4	− 0.0079	0.0026	− 0.0154	− 0.0227	0.0020
5	− 0.0146	0.0024	− 0.0082	− 0.0261	0.0100
6	− 0.0049	0.0009	− 0.0097	− 0.0210	0.0118
7	− 0.0080	0.0023	− 0.0059	− 0.0220	0.0145
8	− 0.0008	0.0010	− 0.0084	− 0.0191	0.0132
9	− 0.0026	0.0011	− 0.0062	− 0.0206	0.0133
10	0.0025	− 0.0001	− 0.0074	− 0.0181	0.0119

Source: Authors' Computations.

*ln* natural logarithm, *FDPRD* food production, *CO2PC* carbon dioxide emissions, *ENUPC* nonrenewable energy per capita, *CR* domestic credit by banks, *RUGR* rural population growth, *ARL* arable land.

**Figure 1:**
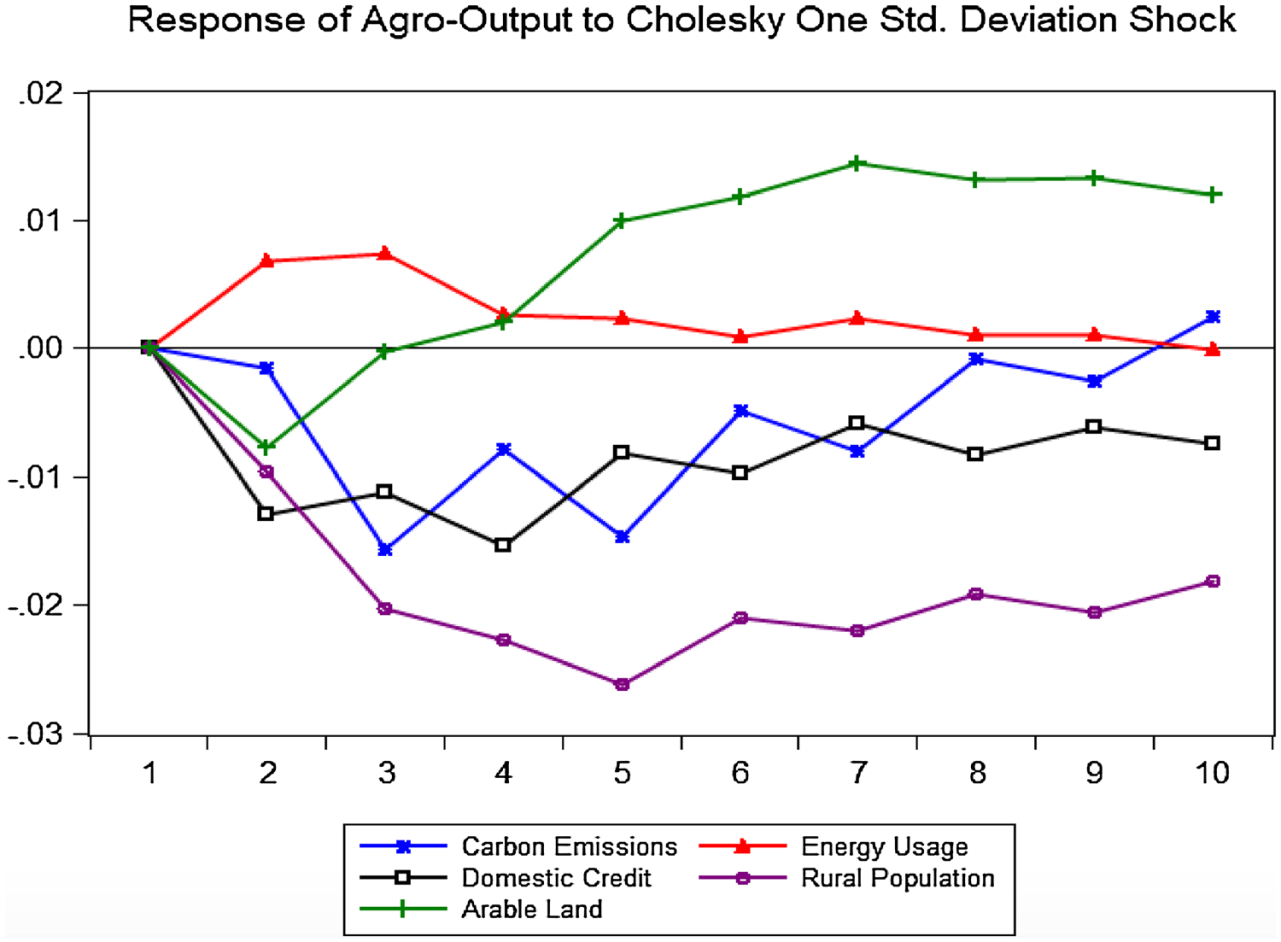
Response of agro-productivity Cholesky one standard deviation shocks. *Source*: Authors’.

Analysis from the IRFs is similar to those obtained from the Johansen cointegration long-run results. Over the 10-year horizon, agro-productivity decreases from a one-standard positive shock to carbon emissions, credit to the agricultural sector and rural population. Similarly, agro-productivity responds positively to a one-standard deviation shock from non-renewable energy use and arable land. Figure 1 shows the analytical explanations presented in Table 6.

## Conclusion and policy recommendations

This study revisits the 2030 United Nations Sustainable Development Goal (SDG) 2 which aims to “*end hunger, achieve food security, improve nutrition and promote sustainable agriculture*” by highlighting the impact of environmental degradation (proxied by carbon emissions) and non-renewable energy on agro-productivity in Nigeria. Using annual time series data from 1980 to 2018, the engagement of the Johansen cointegration and impulse response functions within the vector autoregressive (VAR) framework we provide evidence that: (1) carbon emissions is a significant but consistent negative predictor while non-renewable energy use is a significant and consistent positive predictor of agro-productivity; (2) domestic credit and rural population show negative influence; while (3) arable land increases agro-productivity. These results align with a priori expectations.

Given our findings, the devastating impact of carbon emissions on agriculture cannot ignored otherwise the attainment of SGD 2 will be threatened. Also, being the 17th largest emitter of carbon and with an economy that is weakly-dependent on agriculture, Nigeria must take urgent steps to curb emissions. To this end, the study recommends the following:

1. There must be strict regulatory measure in place to curb the amount of carbon dioxide emissions.
2. More potentials on the usage of non-renewable energy sources must be explored.
3. The Central Bank of Nigeria must re-adjust lending mechanisms to the agrarian sector through the setting up of “special credits” at zero lending rates or initiate credits with longer moratorium.
4. The rural communities must be adequately equipped with basic infrastructures to discourage migration to the cities.
5. More land allocated for agricultural purposes.

For further research, the impact of ecological footprint as a threat to food sustainability in Nigeria may be taken up, given data availability.

## Data Availability

The datasets used and/or analysed during the current study are available from the corresponding author on reasonable request.
